# Parity of esteem within the biopsychosocial model: is psychiatry still a psychological profession?

**DOI:** 10.1192/bjb.2023.62

**Published:** 2023-12

**Authors:** Jo O'Reilly, Rachel Gibbons, Simon Heyland, Jessica Yakeley

**Affiliations:** 1 Camden and Islington NHS Foundation Trust, London, UK; 2 Royal College of Psychiatrists, London, UK; 3 Birmingham and Solihull Mental Health NHS Foundation Trust, Birmingham, UK; 4 Tavistock and Portman NHS Foundation Trust, London, UK

**Keywords:** Formulation, professional identity of psychiatrists, relational care, psychotherapeutic psychiatry, biopsychosocial approach to psychiatry

## Abstract

In recent years, the Royal College of Psychiatrists has been engaged in activities to ensure parity of esteem for mental health within the National Health Service, seeking to bring resources and services more in line with those available for physical health conditions. Central to this has been the promotion of psychiatry as a profession that takes a biopsychosocial approach, considering all aspects of the patient's presentation and history in the understanding and treatment of mental disorders. However, there has been a drift away from considering the psychological aspects of the patient's difficulties in recent years. This potentially has profoundly negative consequences for clinical care, training, workforce retention and the perception of our identity as psychiatrists by our colleagues, our patients and the general public. This editorial describes this issue, considers its causes and suggests potential remedies. It arises from an overarching strategy originating in the Royal College of Psychiatrists Medical Psychotherapy Faculty to ensure parity of esteem for the psychological within the biopsychosocial model.

## The drift away from the psychological

The Royal College of Psychiatrists has rightly campaigned for parity of esteem between physical and mental health among our partners in healthcare provision, politicians and funding bodies.^1^ The recently introduced Health and Care Act 2022^2^ is a further opportunity to improve mental healthcare in the UK. However, we also need to have the capacity to look inside psychiatry, not just at our place at the National Health Service table, and reflect on what state our profession is in. All medical specialties talk about the importance of taking a biopsychosocial approach to patient care. However, a sober assessment would be that such a holistic view has not been fully adopted within many specialties, including psychiatry.

There are concerning indications that our profession no longer prioritises the vital importance of apprehending the biological, psychological and social aspects of our patients’ difficulties and the broad range of interventions needed to help them. Psychiatry has pivoted away from psychological understanding, psychological approaches and psychological research. This has affected our perception of ourselves as psychiatrists, as well as how we are perceived by the public. It impoverishes us professionally and risks making psychiatry less attractive as a career. The psychological and social contributions to the biopsychosocial model of psychiatry have been downgraded and are now in danger of being lost in favour of a solely ‘bio’ model. These concerns are shared in many areas of the psychiatric and mental health community, including suicide prevention.^3–5^

**Fig. 1:**
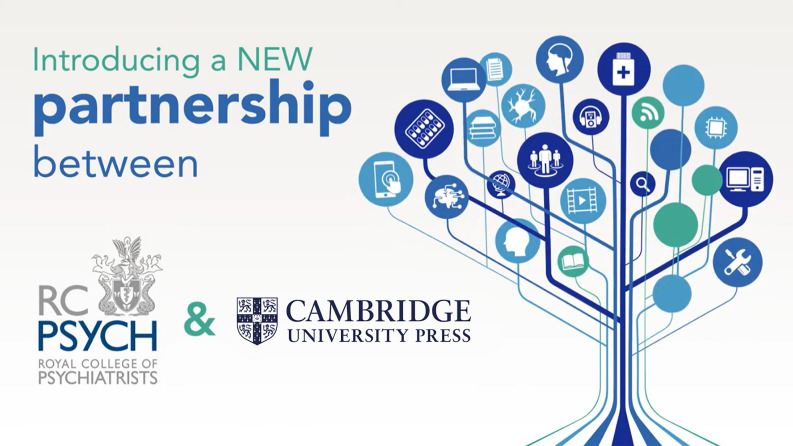
The ‘Tree of Knowledge’ in psychiatry (Cambridge University Press).

This external view of psychiatry as non-psychological is mirrored in our outward-facing communications from the College itself. The graphic for publicity about Royal College of Psychiatrists publications (Fig. 1) is a tree diagram depicting three brains, a neuron, some books, electronic devices, figures standing in an arrowhead formation, a packet of pills and even a screwdriver. There is nothing to represent people relating to or talking with each other.

From this, it would appear that psychiatrists are now seen as less and less interested in the psychological aspects of patient care. Psychologists, counsellors and psychotherapists have become the ‘go-to’ professionals for taking feelings seriously and providing an understanding of psychological distress. The profession also makes much of its pivot towards neuroscience – a discipline which should be seen as not in opposition to but complementary to the psychological understanding of mental illness.^5^

This overly biological vision of psychiatry ignores the pioneering part that psychiatrists have played in the development of psychological interventions and evidence-based treatment modalities such as cognitive therapy,^6^ developed by Aaron Beck; transference-focused psychotherapy,^7^ developed by Otto Kernberg; mentalisation-based treatment,^8^ developed by Anthony Bateman; and psychodynamic interpersonal therapy,^9^ developed by Bob Hobson.

The cause of this drift is likely to be multifactorial, and to some extent it is due to local factors – many consultant psychiatrist jobs are very pressurised, leaving little time for reflection on the complex and multi-faceted factors underlying the presentation of individual patients. Arguably, it is a false premise to regard taking time to think as reducing resources for other more necessary activities, rather than viewing a fully comprehensive case formulation from the outset as allowing more realistic and effective use of resources. However, to a significant extent, we think there are other factors at play that also require attention.

## The dangers of this drift

### Increasing the risk to patients

The capacity to engage psychologically with patients in distress is the basis of therapeutic care. If we do not have clear internal frameworks about psychological functioning to guide us in this work and confidence that developing this understanding is a key part of treatment, we profoundly reduce our capacity to provide meaningful care for our patients. There is a strong evidence-based demand from the suicide prevention community for psychiatric services to move away from tick-box engagement with patients towards dynamic clinical formulation and therapeutic engagement. Research has demonstrated the limits of risk assessment tools as predictors of risk and shown that this type of clinical contact can inflame those in distress. By contrast, a containing clinical encounter which seeks to understand and make sense of the distress based on a biopsychosocial formulation significantly reduces the risk of further self-harm.^10^

### Concrete management of the patient population, resulting in the ‘revolving door’

Clinicians and teams carry out multiple assessments of patients’ presenting symptoms and risk, and discharge from wards or clinical teams may be planned once these issues seem less acute, only for the patient to be repeatedly re-referred as the underlying issues have not been addressed. The sheer weight of referrals can mean that teams have no time to carry out more comprehensive formulation-based assessments to understand the underlying factors driving the presentation to services at this time. There is a common feeling that consultants spend much of their time ‘firefighting’ and are overwhelmed with workload pressures, with no time to think more psychologically about their patients. In the longer term, this is unlikely to lead to effective use of clinical resources.

Without formulation, by which we mean biopsychosocial formulation, we fail our patients. We start to see them as diagnoses, assessable through the use of questionnaires, interviews based on symptom-gathering and tick boxes. We lose the ‘thick description’ that makes our specialty what it is. Why has this person become depressed now? What is the link between the loss of their daughter 8 years ago, their son leaving home and their symptoms? How is their expression and communication of distress affected by their early history of abandonment, abuse, parental neglect? We need to ask ourselves who would want to be a psychiatrist if we strip away the meanings and connections in the stories our patients bring us.

This is particularly important for complex patients with particularly complicated social backgrounds and comorbidities. This group of patients is most reliant on professionals holding the different aspects of their problems in mind, rather than splitting them off into different professional groupings or a range of specialist teams for different symptoms. This includes medically unexplained symptoms and factitious disorders, complex trauma and personality disorders, complex eating disorders, and comorbid conditions alongside psychotic disorders, which are increasingly common in routine psychiatric practice. The most complex patients are the ones that most need professionals who are able to hold their biological, psychological and social needs in mind at the same time. Psychiatrists are the only profession trained to do this, and in fact historically this was a core part of our role. This very complex group forms an increasing part of consultant psychiatrists’ workload.

Increasing workload pressures, difficulty in retaining staff and increasing specialisation of psychiatric teams have all contributed to real challenges in providing continuity of care to our patients. This can present significant challenges, as patients may experience multiple short-term relationships with different clinical teams, which may repeat experiences of disrupted attachments, a bewildering array of opinions and advice, and loss of caring relationships they can rely on. The importance of communication and effective case formulation involving our patients and their carers and loved ones, with care based on a holistic and shared understanding of their difficulties, is of even greater importance when working under these conditions if care itself is not to become fragmented.

### Making psychiatry less attractive to medical students

Interest in the psychological is an important part of what draws trainees into our specialty.^11^ ‘Choose Psychiatry’,^12^ the College's campaign aiming to encourage more medical students and doctors to specialise in psychiatry, includes establishing medical student psychotherapy experience and Balint groups within medical schools across the UK, which evidence has shown are major draws into psychiatry. Students who participate in a psychotherapy scheme (in which they are involved in delivering weekly psychotherapy for a patient under supervision, as part of learning about emotional aspects of the doctor–patient relationship) are significantly more likely to choose psychiatry as a career, even those who were not previously interested in psychiatry.^13^ Doctors thinking of joining the specialty still think of psychiatrists as mostly listening and talking to their patients towards developing a deeper understanding of their difficulties and histories. We lose this at our peril.

### Deskilling our trainees in formulation

Significant progress has been made in recent years to reinforce the place of psychological training within core training, with all trainees now required to participate in a Balint group and treat a minimum of two psychotherapy cases. The Balint group is intended to embed reflective practice as an essential component of a psychiatrist's working life, deepening clinical understanding, enabling processing of the emotional impact of the work and supporting team functioning.

The psychological part of the biopsychosocial model of psychiatry is not, however, just about becoming reflective practitioners^14^ and learning how to deliver different forms of psychotherapy. It is also about developing a biopsychosocial formulation as the basis of clinical care for all patients presenting to mental health services.

Loss of the long case in the examination for membership of the Royal College of Psychiatrists means that a key driver for developing case formulation skills has been removed. Although trainees are expected to formulate in a biopsychosocial way, anecdotally it is evident that this skill is becoming a lost art in UK psychiatry. In case presentations and academic meetings, it is now common to see trainees and senior colleagues move from the presentation of a patient's history to differential diagnosis, with no reference to a biopsychosocial formulation beforehand. It is as if the gathering of information is in the service of eliciting symptoms as part of a checklist and then rushing to a model or psychiatry in which a diagnosis is made and treatment prescribed. In taking a psychiatric history, we are privileged in the richness of the material we hear, full of meaning about someone's life. Yet it seems that trainees are no longer trained to use the information gleaned about their patients’ early development, relationship and attachment patterns, losses, and traumatic and other formative experiences.

A comprehensive case formulation is what enables us to understand our patients’ presentations and the challenges routinely faced in everyday psychiatry. The psychological component of a biopsychosocial approach supports an attitude of open curiosity about the person in need of mental health services, their emotions and thoughts, and the experiences and relationships most significant to them. It can also shed invaluable light on someone's vulnerability to psychiatric breakdown, what their symptoms may mean, the psychological and relational factors that may support or inhibit recovery, treatment non-compliance, and the reasons underpinning risky and self-destructive behaviours. The practice of psychiatry is a complex task; we need a fully biopsychosocial approach that seeks to understand the underlying issues behind our patient's presentations, in order to provide care on a truly holistic, meaning-making, relational and person-centred basis.

We are facing considerable challenges with recruitment and retention of the workforce within psychiatry. Although most specialisms within psychiatry have vacancies in higher training posts, fill rates for dual training in medical psychotherapy aligned with adult psychiatry are 100%. These posts are oversubscribed, strongly suggesting that trainees wish to develop into psychotherapeutically informed practitioners able to apply these skills across a wide range of psychiatric settings.

### Deskilling consultants in formulation

If we don't train our workforce in comprehensive case formulation, we produce consultants who similarly lack these skills. There is no reason to think that such skills are no longer necessary in psychiatric practice – psychopharmacology and brain science have not cured mental illness. Psychological models offer vital ways to understand why our patients break down in the manner they do, the potential triggers for this, the relationship templates learned in early life, which may become manifest in the relationships created with clinical teams, and psychological aspects of treatment resistance – all of which may present management challenges in everyday psychiatry and hence require understanding in the service of providing compassionate clinical care and effective management planning.

### Recommending psychological treatments

As psychiatrists, we are generally much less aware of advances in psychological approaches than we are of those in pharmacological approaches. Yet arguably the majority of advances in the past 30 years have been in the former. Many psychiatrists treat psychological interventions as black boxes in which they have little expertise or understanding and refer for (effectively) a second opinion rather than accurately ‘prescribing’ a psychological intervention. There is a good case for psychiatrists being as knowledgeable about psychological interventions as they are about pharmacological interventions. For example, when a patient presents with post-traumatic stress disorder, a psychiatrist would consider prescribing an antidepressant and consider referral for psychological treatment of some sort. This tends to lead to an additional assessment, and to dissatisfaction, as patients are encouraged to think of the psychological treatment as belonging ‘somewhere else’. A better way would be for the psychiatrist to feel confident to engage in an active discussion with the patient about psychological treatments which may help, having built a shared formulation of their difficulties and understanding of their most pressing concerns. Interventions such as trauma-based cognitive–behavioural therapy, eye movement desensitisation and reprocessing therapy, or psychodynamic psychotherapy could then be recommended as part of a comprehensive package of care in which psychological interventions and pharmacological interventions are managed in one place. Why should psychiatrists be less aware of the evidence for psychological treatments than that for pharmacological or physical treatments?

### Providing psychological treatments

We train psychiatrists to be competent to deliver psychological treatments. A few (other than medical psychotherapists) continue to do this as consultants, but it is often difficult to timetable this into job plans. Trusts tend to see psychological treatments as best provided by other professions. We think this is because of the shifting perception of what psychiatrists do – assessing, diagnosing, prescribing, applying the Mental Health Act, and managing and holding responsibility for risk. This diminishes our role and the breadth of our training to function as psychologically informed practitioners. Colleagues who can deliver psychological interventions are enthusiastic about being able to do so. It is a mistake to assume that psychiatrists will not deliver expert psychological treatments. We have let this slip.

### Psychological research

There has been an explosion in research in both novel and established methods of psychological treatment. In stark contrast to pharmacological research, these novel methods are producing positive results in areas for which there was previously little or no treatment, for example, personality disorder. Moreover, there is a wealth of cross-disciplinary studies linking research in psychological therapies to related fields such as neuroscience, experimental psychology and developmental research.^15^ Are most psychiatrists aware of this? It is hard to say, but a cursory glance at the schedule for the 2022 RCPsych International Congress will tell you that the extraordinary developments in psychological and psychotherapeutic approaches are having difficulty reaching the surface. A brief survey of the conference programme identifies 32 sessions on pharmacological and neuroscientific topics (mostly symposia composed of three or four sessions); 13 broadly socially focused sessions, on subjects such as the aftermath of Covid, inequality, the social determinants of mental illness and the impact of social media; and a number of sessions that don't fit into biological, psychological or social categories, such as risk assessment and phenomenology. However, there were only seven sessions on psychological approaches and understanding. There was not, as far as we could find, a single session on developments in psychological treatments. Does this accurately represent progress in practice and how we how we think about psychiatry?

## Debate and controversies: past and present

Arguments and different views about the relative contributions of biological, psychological and social factors to the development and maintenance of mental illness are not new to our profession. Advances in biological sciences such as epigenetics, neurobiology and attachment theory point to a complex interplay between psychological factors, early relationships and the influence of the social environment on gene expression and brain development; the science supports the importance of a biopsychosocial approach to psychiatry. As psychiatrists we are acutely aware in our daily work of the impact of childhood adversity and social disadvantage on so many of our patients. Yet the drift away from holistic case formulation and an insufficient focus on psychological, relational and social factors continue in everyday psychiatric care and in our academic and training activities. There is an urgent need to develop these concerns into real changes in how we practise in our daily work and to ensure psychiatrists are trained and supported to do so. Craddock et al^16^ describe the risks to our identity as psychiatrists and to patient care if we do not clearly articulate our expertise, with a risk of a ‘creeping devaluation’ of our central role in patient care. Although the emphasis of their article differs from ours, the argument about loss of our identity and the need to represent and to debate our professional skills robustly remains. Unless we can stand up for our breadth of training and demonstrate a multifaceted approach to clinical care, we risk colluding with a gradual erosion of our skillset and becoming diminished as a profession, to the impoverishment of clinical care. Counterarguments may include that different professionals within a multidisciplinary team may be better equipped to contribute psychological thinking in clinical care; we would argue that in our leadership roles, holding clinical responsibility in care, it is vital that as psychiatrists we are able to demonstrate skill and confidence in formulating our patient's difficulties and practising as psychologically skilled practitioners.

## Next steps

We should take the view that psychiatry is a psychological profession; that the psychological enriches the experience of psychiatrists and benefits patients; that neglecting the psychological within our work supports critics who view psychiatrists as pill-pushers and locker-uppers; and that if psychiatry doesn't fill the psychological space, others will, leaving patients with serious and complex mental illnesses without access to the high-level integrated care that they need. We therefore need to work to secure parity for the psychological within the biopsychosocial model by embedding psychotherapeutic approaches in psychiatric practice. This would involve a range of measures.

- Recognising that our role is to take leadership in biopsychosocial formulation in our teams and for our patients and demonstrating that case formulation provides the bedrock for decisions about clinical care within clinical meetings, case discussions, academic programmes and supervision.
- Actively supporting the provision of and attendance at high-quality reflective practice as routine within psychiatry to further develop the psychological understanding of complex clinical presentations and to process the emotional impact of the work.
- Ensuring that psychiatrists are trained to develop comprehensive biopsychosocial case formulation as the basis of clinical understanding and management for all cases; the new curricula embed complex case formulation within training at both core and higher levels, yet it is not currently clear how this will be assessed.
- Ensuring that psychiatrists have the skills and confidence to prescribe and apply psychological treatments in their practice.
- Using appraisal and job planning for consultants to advocate for and support the maintenance of higher-level psychotherapeutic competencies in consultants and ensuring that continuing professional development activities cover a broad range of approaches to psychiatry.
- Working with patients and carers to establish the expectation that psychological approaches are embedded in psychiatry services.
- Incorporating this message into the College's vision of itself and its strategic communications.

Medical psychotherapists can take a lead on some aspects of this, but a psychological approach to our profession should be owned by all psychiatrists regardless of subspecialty. The biopsychosocial model should be truly equitable across all three parts: bias towards the biological, sociological or psychological moves us away from the real complexity of psychiatric presentations and from fully seeing the person behind the symptoms, and it deprives us of the opportunity to use complementary explanations and treatments in a mutually illuminating, creative and helpful manner in clinical care.

## About the authors

**Jo O'Reilly** is a consultant psychiatrist in Medical Psychotherapy, Camden and Islington NHS Foundation Trust, UK; Chair of the Medical Psychotherapy Faculty Executive Committee, Royal College of Psychiatrists; and a member of the British Psychoanalytic Society. **Rachel Gibbons** is a consultant psychiatrist in Medical Psychotherapy and a group analyst; Vice Chair of the Medical Psychotherapy Faculty Executive Committee, Royal College of Psychiatrists, UK; and a member of the British Psychoanalytic Society. **Simon Heyland** is a consultant psychiatrist in Medical Psychotherapy, Birmingham and Solihull Mental Health NHS Foundation Trust, UK; and a member of the Medical Psychotherapy Faculty Executive Committee, Royal College of Psychiatrists, UK. **Jessica Yakeley** is a consultant psychiatrist in Forensic Psychotherapy; Director of the Portman Clinic, Tavistock and Portman NHS Foundation Trust, UK; a member of the Medical Psychotherapy Faculty, Royal College of Psychiatrists, UK; and a Fellow of the British Psychoanalytic Society.

## References

[1] Royal College of Psychiatrists. Whole-Person Care: From Rhetoric to Reality (Achieving Parity Between Mental and Physical Health). Occasional paper 88. RCPsych, 2012.

[2] *Health and Care Act 2022* (https://www.legislation.gov.uk/ukpga/2022/31/contents/enacted).

[3] Bolton D. Looking forward to a decade of the biopsychosocial model. BJPsych Bull 2022; 46: 228–32. 10.1192/bjb.2022.34 PMC9768524 35781123

[4] Williamson S. The biopsychosocial model: not dead but in need of revival. BJPsych Bull 2022; 46: 232–4. 10.1192/bjb.2022.29 PMC9768512 35678035

[5] Tripathi A, Das A, Kar SK. Biopsychosocial model in contemporary psychiatry: current validity and future prospects. Indian J Psychol Med 2019; 41(6): 582–5. 31772447 10.4103/IJPSYM.IJPSYM_314_19 PMC6875848

[6] Beck AT. Cognitive Therapy and the Emotional Disorders. International Universities Press, Inc., 1975.

[7] Clarkin JF, Yeomans F, Kernberg OF. Psychotherapy for Borderline Personality: Focusing on Object Relations. Wiley, 2006.

[8] Bateman A, Fonagy P. Psychotherapy for Borderline Personality Disorder: Mentalization-Based Treatment. Oxford University Press, 2004.

[9] Barkham M, Guthrie E, Hardy G, Margison F. Psychodynamic-Interpersonal Therapy: A Conversational Model. Sage, 2017.

[10] Kapur N, Steeg S, Haigh R, Bergen H, Hawton K, Ness J, et al. Does clinical management improve outcomes following self-harm? Results from the mutlicentre study of self-harm in England. PLoS One 2013; 8(8): e70434. 23936430 10.1371/journal.pone.0070434 PMC3731259

[11] Goldenberg MN, Williams DK, Spollen JJ. Stability of and factors related to medical student specialty choice of psychiatry. Am J Psychiatry 2017; 174: 859–66. 28618855 10.1176/appi.ajp.2017.17020159

[12] Royal College of Psychiatrists. *Choose Psychiatry: Guidance for Medical Schools*. RCPsych, 2019 (https://www.rcpsych.ac.uk/become-a-psychiatrist/choosepsychiatry/choose-psychiatry-guidance-for-medical-schools).

[13] Yakeley JW, Shoenberg P, Heady A. Who wants to do psychiatry? The influence of a student psychotherapy scheme – a ten year retrospective study. Psychiatr Bull 2004; 28: 208–12.

[14] General Medical Council. *The Reflective Practitioner – Guidance for Doctors and Medical Students*. GMC, 2021 (https://www.gmc-uk.org/-/media/documents/dc11703-pol-w-the-reflective-practioner-guidance-20210112_pdf-78479611.pdf).

[15] Holmes EA, Ghaderi A, Harmer CJ, Ramchandani PG, Cuijpers P, Morrison AP, et al. The Lancet Psychiatry Commission on psychological treatments research in tomorrow's science. Lancet Psychiatry 2018; 5: 237–86. 29482764 10.1016/S2215-0366(17)30513-8

[16] Craddock N, et al. Wake-up call for British psychiatry. Br J Psychiatry 2008; 193: 511–2. 10.1192/bjp.bp.108.053561 18700211

